# Suppressed basal melting in the eastern Thwaites Glacier grounding zone

**DOI:** 10.1038/s41586-022-05586-0

**Published:** 2023-02-15

**Authors:** Peter E. D. Davis, Keith W. Nicholls, David M. Holland, Britney E. Schmidt, Peter Washam, Kiya L. Riverman, Robert J. Arthern, Irena Vaňková, Clare Eayrs, James A. Smith, Paul G. D. Anker, Andrew D. Mullen, Daniel Dichek, Justin D. Lawrence, Matthew M. Meister, Elisabeth Clyne, Aurora Basinski-Ferris, Eric Rignot, Bastien Y. Queste, Lars Boehme, Karen J. Heywood, Sridhar Anandakrishnan, Keith Makinson

**Affiliations:** 1 British Antarctic Survey, Cambridge, UK; 2 Courant Institute of Mathematical Sciences, New York University, New York, NY USA; 3 Center for Global Sea Level Change, New York University Abu Dhabi, Abu Dhabi, UAE; 4 Department of Astronomy, Cornell University, Ithaca, NY USA; 5 Department of Environmental Studies, University of Portland, Portland, OR USA; 6 College of Earth, Ocean, and Atmospheric Sciences, Oregon State University, Corvallis, OR USA; 7 Georgia Institute of Technology, Atlanta, GA USA; 8 Department of Geosciences, Pennsylvania State University, State College, PA USA; 9 Environmental Studies, Lewis & Clark College, Portland, OR USA; 10 Department of Earth System Science, University of California, Irvine, Irvine, CA USA; 11 Jet Propulsion Laboratory, California Institute of Technology, Pasadena, CA USA; 12 Department of Marine Sciences, University of Gothenburg, Gothenburg, Sweden; 13 Scottish Oceans Institute, University of St Andrews, St. Andrews, UK; 14 Centre for Ocean and Atmospheric Sciences, School of Environmental Sciences, University of East Anglia, Norwich, UK

**Keywords:** Physical oceanography, Physical oceanography, Cryospheric science, Cryospheric science

## Abstract

Thwaites Glacier is one of the fastest-changing ice–ocean systems in Antarctica^1–3^. Much of the ice sheet within the catchment of Thwaites Glacier is grounded below sea level on bedrock that deepens inland^4^, making it susceptible to rapid and irreversible ice loss that could raise the global sea level by more than half a metre^2,3,5^. The rate and extent of ice loss, and whether it proceeds irreversibly, are set by the ocean conditions and basal melting within the grounding-zone region where Thwaites Glacier first goes afloat^3,6^, both of which are largely unknown. Here we show—using observations from a hot-water-drilled access hole—that the grounding zone of Thwaites Eastern Ice Shelf (TEIS) is characterized by a warm and highly stable water column with temperatures substantially higher than the in situ freezing point. Despite these warm conditions, low current speeds and strong density stratification in the ice–ocean boundary layer actively restrict the vertical mixing of heat towards the ice base^7,8^, resulting in strongly suppressed basal melting. Our results demonstrate that the canonical model of ice-shelf basal melting used to generate sea-level projections cannot reproduce observed melt rates beneath this critically important glacier, and that rapid and possibly unstable grounding-line retreat may be associated with relatively modest basal melt rates.

## Abstract (web summary)

Despite observations from a hot-water-drilled access hole showing warm ocean waters beneath Thwaites Glacier Eastern Ice Shelf, the basal melt rate is strongly suppressed due to the low current speeds and strong density stratification.

## Main

The response of the marine-based West Antarctic Ice Sheet (WAIS) to a warming climate contributes substantial uncertainty to twenty-first century sea-level projections^9^. The evolution of the ice sheet is dynamically linked to the fate of the floating ice shelves found at its seaward margin^10^. By exerting a resistive force at the grounding line where the ice sheet first goes afloat, ice-shelf buttressing helps control the flow of grounded ice into the ocean^11^. Over recent decades, elevated ocean-driven basal melting has triggered rapid thinning of many West Antarctic ice shelves^12^, reducing the strength of ice-shelf buttressing^11^. The rate of ice-shelf mass loss has increased by 70% between 1994 and 2012 (ref. ^12^), precipitating a shift towards faster drainage of grounded ice into the ocean^13^. Several major grounding lines in the Amundsen Sea sector have retreated rapidly inland^14^, raising the possibility of an unstable collapse of the WAIS^15^.

**Fig. 1:**
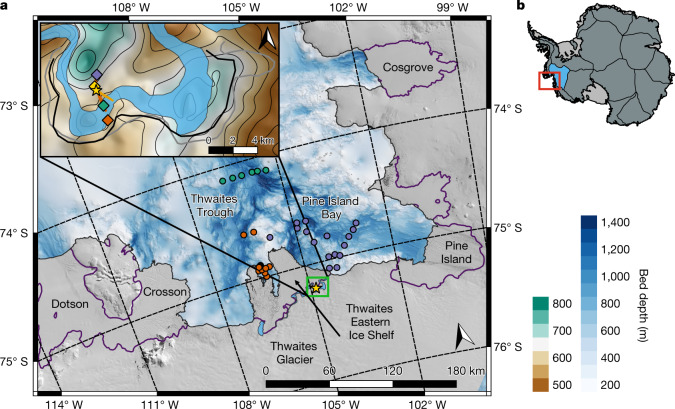
Map of Thwaites Glacier and location of the observations used in this study. **a**, Landsat 8 satellite image of Thwaites Glacier and the location of the hot-water-drilled access hole (yellow star; 75.207° S, 104.825° W) in the grounding-zone ‘butterfly’ region of TEIS (inset map). Blue-coloured contours with hillshade show bed depth in the Amundsen Sea from ship-based survey^49^ and BedMachine^5^. The lilac, green and orange dots show the location of 2019–2020 ship-based CTD profiles from the International Thwaites Glacier Collaboration TARSAN project. The coastline (black) and grounding line (purple) are from the SCAR Antarctic Digital Database^50^. The inset map shows the detail of the grounding-zone butterfly region. Green–brown-coloured contours with hillshade show bed depth from BedMachine^5^. The blue-shaded area shows the location of the 2016–2017 grounding-zone region^18^, whereas the solid black and grey lines show the position of the grounding line in 2019 and 2021, respectively. Green, purple, orange and yellow diamonds show the location of ApRES instruments measuring the basal melt rate, in addition to the ApRES located at the hot-water-drilled access hole (yellow star). The red (T1) and orange (T2) lines show the transects taken by the Icefin remotely operated underwater vehicle. **b**, Overview of Antarctica using data from MEaSUREs Antarctic Boundaries^51^ with the location of Thwaites Glacier shown by the red box. Thin black lines delineate the main ice-sheet drainage basins^52^, with the Thwaites drainage basin highlighted in blue. Figure 1 was created with the QGIS Geographic Information System.

Nowhere are these processes more apparent and potentially serious than at Thwaites Glacier, which drains about 10% of the WAIS^1,16^ (Fig. 1). Thwaites is largely grounded below sea level on a retrograde bed^4^ (that is, a bed that deepens inland) and is particularly susceptible to marine ice-sheet instabilities^2,3^. Its grounding line has retreated 14 km inland since the late 1990s^17^ and, in some regions, is retreating by up to 1.2 km per year at present (ref. ^18^). Thwaites may have already entered a state of rapid and irreversible ice loss^3^, and its complete collapse within centuries would contribute 65 cm to the global sea level^5^. A full destabilization of the main glaciers in the Amundsen Sea sector would contribute 3 m to the global sea level over thousands of years^19^. The rate and extent of ice loss from Thwaites Glacier, and whether it proceeds irreversibly, is highly sensitive to the poorly understood ocean conditions and basal melt rate in the constantly evolving grounding-zone region^6^.

Here, to our knowledge, we present the first observations from the Thwaites Glacier grounding zone. A hot-water-drilled access hole was made through 587 m of ice approximately 1.5–2.0 km downstream of the present-day grounding line (Fig. 1) in the relatively accessible ‘butterfly’ region of TEIS. A borehole-deployable conductivity, temperature and depth (CTD) profiler was used to sample the hydrographic structure of the 54-m-deep water column, while the Icefin remotely operated underwater vehicle measured the spatial variability in ocean conditions all the way to the grounding line^20^. Long-term basal melt rates at five different sites (Fig. 1) were measured using Autonomous phase-sensitive Radio-Echo-Sounder^21^ (ApRES) and an oceanographic mooring consisting of a current meter and a temperature–conductivity sensor was deployed 1.5 m beneath the ice-shelf base to monitor the temporal evolution of ocean properties. Wider oceanographic context is provided by ship-based CTD profiles from 2019 and 2020 (ref. ^22^) (Fig. 1).

## Water-column structure and hydrography

**Fig. 2:**
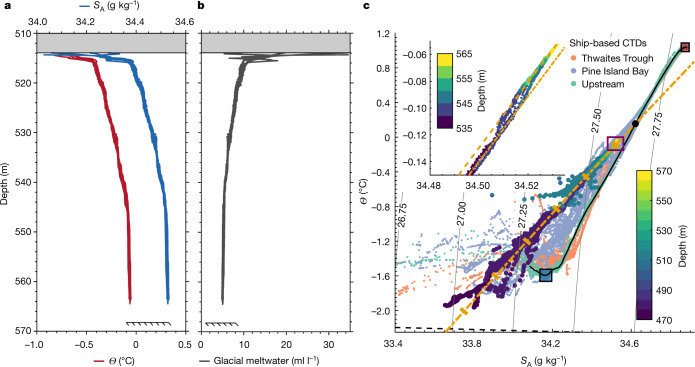
Hydrography and meltwater content beneath TEIS. **a**,**b**, Vertical profiles of conservative temperature (*Θ*; red) and absolute salinity (*S*_A_; blue) (**a**) and glacial meltwater content (grey) (**b**) collected over 4 days (9 to 12 January 2020) in the grounding-zone region of Thwaites Glacier (yellow star in Fig. 1). The ice base is indicated by the shaded grey box and the seabed is indicated by the slash-backed line. **c**, *Θ*–*S*_A_ diagram with *σ*_0_ (density) contours for the grounding-zone CTD and Icefin data (large dots coloured by depth) and the ship-based CTD data from the International Thwaites Glacier Collaboration TARSAN project (small dots coloured by location: orange for Thwaites Trough, purple for Pine Island Bay and green for upstream that match the colours used to indicate their location in Fig. 1). The solid black line indicates the ambient mCDW–WW thermocline. The dot-dashed orange line indicates the meltwater mixing line that characterizes the grounding-zone data. The large black dot indicates where this meltwater mixing line intersects the ambient mCDW–WW thermocline. The thick orange dashes on the meltwater mixing line indicate 5 ml l^−1^ intervals in glacial meltwater content, starting at 0 ml l^−1^ at the large black dot. The dashed black line indicates the in situ freezing temperature as a function of salinity at the grounding line. The red and blue boxes with black outline indicate the range of *Θ* and *S*_A_ values of the mCDW and WW endmembers. The inset axes in **c** show the *Θ*–*S*_A_ relationship coloured by depth (note the different colour scale) for the CTD data from the well-mixed benthic boundary layer (purple box in the main plot). The dashed orange line indicates the slightly warmer meltwater mixing line that characterizes the data from this region of the water column.

**Extended Data Fig. 1:**
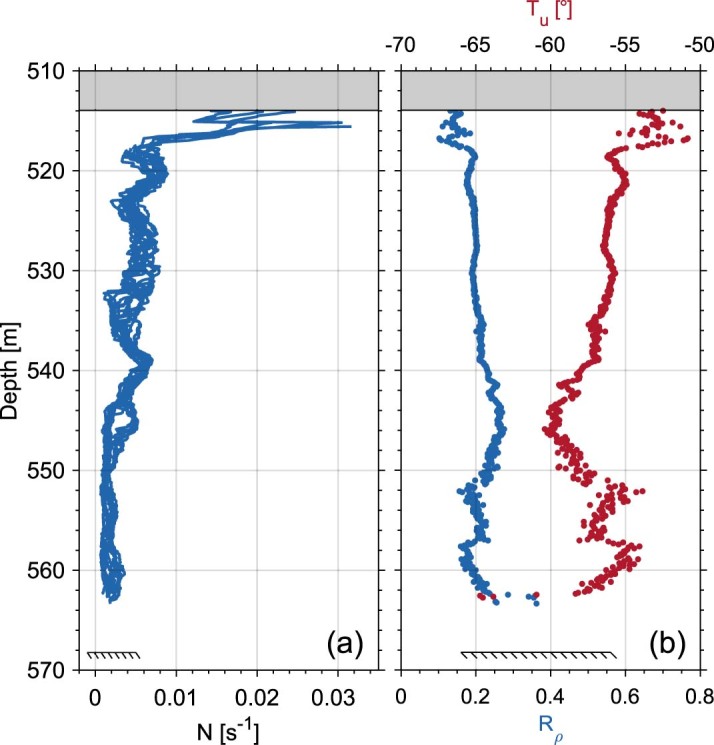
Vertical profiles of buoyancy frequency, density ratio and Turner angle. **a**,**b**, Individual vertical profiles of buoyancy frequency (*N*) (**a**) and average vertical profile of the density ratio (blue) and Turner angle (red; see Methods) (**b**) collected over 4 days (9 to 12 January 2020) in the grounding-zone region of Thwaites Glacier (yellow star in Fig. 1). The ice base is indicated by the shaded grey box and the seabed is indicated by the slash-backed line.

The grounding zone is characterized by warm and salty water at depth, with cooler and fresher water at the ice base (Fig. 2a). Thermal driving near the ice–ocean interface (a key parameter for controlling basal melting; see Methods) reaches 1.54 °C, similar to beneath the Pine Island Ice Shelf^23^. A highly salt-stratified basal boundary layer is seen within 2 m of the ice–ocean boundary, where the sharp gradient in absolute salinity (*S*_A_) creates a strong barrier to vertical mixing (Extended Data Fig. 1a). Although density in the polar regions is set by salinity and thus the grounding-zone water column is stably stratified (Fig. 2c), the vertical gradient of conservative temperature (*Θ*) is unstable with respect to density (that is, cold water lies above warm water) and the water column might be susceptible to diffusive convection. Although this double-diffusive process could provide a limited source of energy for vertical mixing^24–26^, with an average density ratio of only 0.2 and a Turner angle of −57°, the temperature gradient is too weak to sustain a thermohaline staircase (Extended Data Fig. 1b; see Methods). Low variability between CTD casts indicates that lateral gradients in temperature and salinity are weak (see Methods).

Basal melting is forced by a single source water mass: modified Circumpolar Deep Water (mCDW)^22^. The Icefin and borehole-based CTD data lie predominantly on a straight line in *Θ*–*S*_A_ space with a gradient of 2.40 ± 0.01 °C (g kg^−1^)^−1^ (Fig. 2c). The gradient is consistent with that expected when glacial meltwater from ocean-driven basal melting mixes with ambient seawater^27^. The properties of the source mCDW can be determined by tracing the meltwater mixing line back to its intersection with the main mCDW–Winter Water (WW) thermocline outside the ice-shelf cavity (Fig. 2c). The source mCDW has a *Θ* value of 0.16 °C and an *S*_A_ value of 34.62 g kg^−1^, with a potential density of 1,027.66 kg m^−3^. mCDW with such density is found at a depth of around 528 m outside the ice-shelf cavity. The mCDW that feeds the grounding zone probably originates from Pine Island Bay; however, we cannot rule out a more northerly source from Thwaites Trough^22,28^ (Fig. 1). In the well-mixed lower layer, the CTD data shift onto a slightly warmer meltwater mixing line (source water *Θ* = 0.18 °C; Fig. 2c, inset), indicating that the lower cavity is fed by a slightly warmer mCDW.

Glacial meltwater plays a central role in controlling ocean circulation around Antarctica^29^. At the borehole, glacial meltwater is found throughout the water column, with the concentration exceeding 10 ± 2 ml l^−1^ at the ice base (Fig. 2b; see Methods). The meltwater distribution indicates that ocean water at all depths has interacted with an ice-shelf base, consistent with the narrow water column and proximity to the grounding line. At the grounding line itself, the glacial meltwater concentration observed by Icefin reaches a maximum value of approximately 31 ml l^−1^ (Fig. 2c). This is close to the saturation value of approximately 35 ml l^−1^, at which point *Θ* is at the in situ freezing point and no further basal melting can occur.

## Temporal variability in ocean conditions

**Fig. 3:**
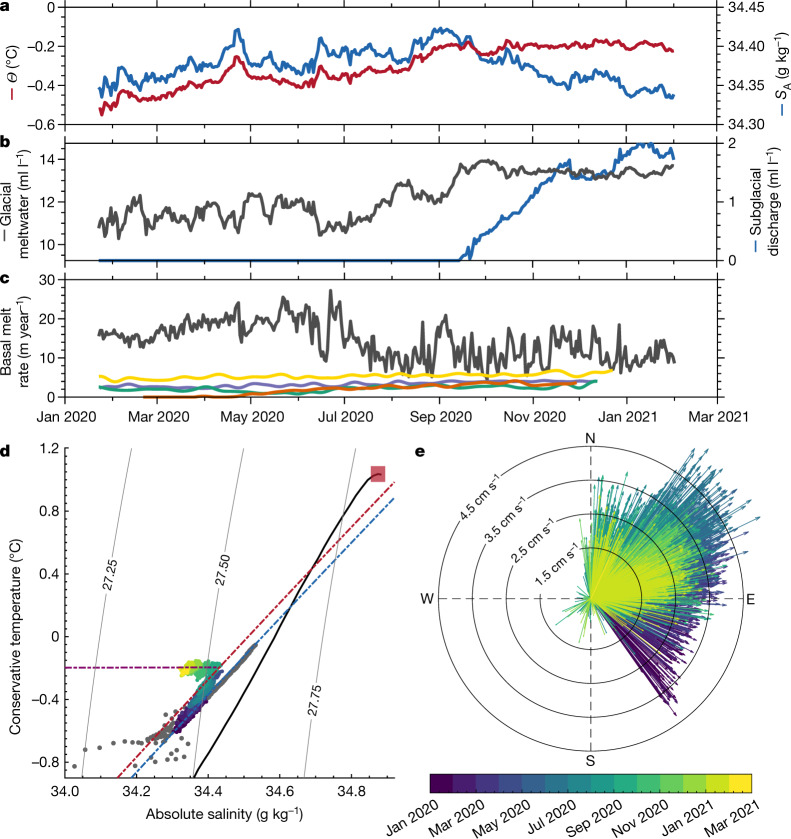
Temporal evolution of hydrographic conditions, meltwater content and basal melt rate. **a**, Daily averaged time series of conservative temperature (*Θ*; red) and absolute salinity (*S*_A_; blue) from the ocean mooring deployed 1.5 m beneath the ice base. **b**, Glacial meltwater (grey) and subglacial runoff (blue) derived from observations of *Θ* and *S*_A_. **c**, Observed ApRES basal melt rate (green, purple, yellow and orange lines) low-pass-filtered with a 15-day cutoff plotted against the basal melt rate estimated from the three-equation melt-rate model (grey line; see Methods). The line colours for the ApRES basal melt-rate time series in **c** match their locations in Fig. 1. **d**, *Θ*–*S*_A_ diagram with *σ*_0_ contours for the time series data in **a** coloured as a function of time. The blue and red dot-dashed lines are meltwater mixing lines that fit the observed data for January 2020 (blue) and August 2020 (red). The purple dot-dashed line is a mixing line between the grounding-zone *Θ* and *S*_A_ values in August 2020 and fresh subglacial runoff. The solid black line indicates the ambient mCDW–WW thermocline from ship-based CTD data (Fig. 2c), whereas the red-shaded box indicates the range of *Θ* and *S*_A_ values of the mCDW endmember. The grey dots show the CTD data from the borehole. **e**, Velocity vectors from the sub-ice current meter coloured as a function of time. Radial contours indicate flow speed in cm s^−1^.

**Fig. 4:**
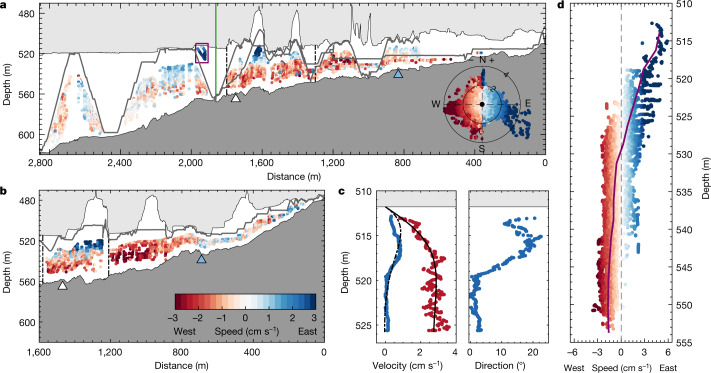
Cross-sections and vertical profiles of current speed and direction. **a**,**b**, Flow speed and direction in the grounding-zone region from an ADCP mounted on the Icefin remotely operated underwater vehicle for transect T1 (**a**) and transect T2 (**b**) (see inset panel in Fig. 1). Individual data points are coloured by flow speed, with blue colours indicating flow to the east (into the page) and red colours indicating flow to the west (out of the page). The vehicle track is indicated by the grey line, with the ice shelf and seabed indicated by the light grey and dark grey patches, respectively. The green line in **a** marks the location of the borehole, and the purple box indicates the region of the water column plotted in **c**. Inset in **a** is geographic velocity vectors coloured by flow speed for the combined data from T1 and T2. Radial contours indicate flow speed in cm s^−1^. Triangles in **a** and **b** mark the location of historic grounding-line locations estimated from satellite interferometry in 2011 (white) and the furthest downstream estimate in 2016 (blue)^18^. **c**, *u* eastward velocity (blue), *v* northward velocity (red) and geographic flow direction within 14 m of the ice base about 2,000 m from the grounding zone along T1 (purple box in panel **a**). The dot-dashed and solid black lines show the *u* (dot-dashed) and *v* (solid) velocity profiles from an analytical model of an under-ice Ekman boundary layer. **d**, Average velocity profile coloured by flow speed for all velocity data between 1,300 m and 1,800 m from the grounding zone along transect T1 (black dot-dashed lines in panel **a**) and between 1,210 m and 1,580 m from the grounding zone along transect T2 (black dot-dashed lines in panel **b**).

**Extended Data Fig. 2:**
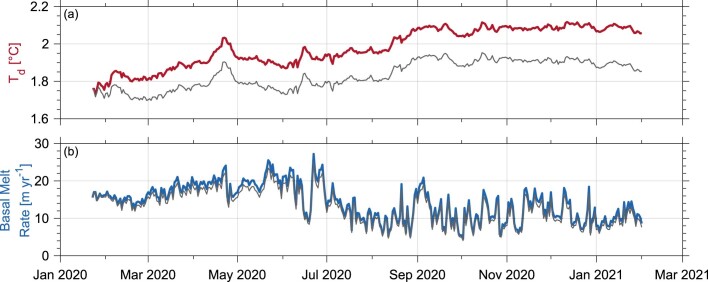
Thermal driving and basal melt rate from the three-equation melt-rate model. **a**,**b**, Daily averaged time series of thermal driving (red) (**a**) and basal melt rate (blue) (**b**) predicted by the three-equation melt-rate model (see Methods). The grey lines in **a** and **b** show the thermal driving and basal melt rate corrected for effects of ice-base recession using the vertical profiles of *Θ* and *S*_A_ from the CTD data (see Methods).

Ocean conditions in the Amundsen Sea vary across a wide range of timescales and affect the ocean properties and basal melt rate beneath the fringing ice shelves^30–32^. Between January and September 2020, the grounding zone became warmer and saltier (Fig. 3a). In *Θ*–*S*_A_ space, the hydrographic properties evolved along a trajectory that lies at an angle to the meltwater mixing line from the CTD profile (Fig. 3d). This trajectory can only be explained by a change in the source water mass. By September 2020, the *Θ* and *S*_A_ values of the mCDW feeding the grounding zone increased to 0.43 °C and 34.69 g kg^−1^, respectively, with a potential density of 1,027.70 kg m^−3^. mCDW with this density is found at a depth of around 584 m outside the ice-shelf cavity. The depth of the seabed and the prograde bedrock slope at the borehole (Fig. 4a) prevents this denser mCDW from reaching the grounding zone directly. Instead, the mCDW–WW thermocline outside the ice-shelf cavity must have shoaled, flooding the grounding zone with increasingly warmer mCDW. Long-timescale variability in thermocline depth is largely controlled by slowly evolving trends in remote wind forcing at the Amundsen Sea continental shelf break^32^. Superimposed on the warming trend are short pulses of warming and cooling (for example, April and June 2020; Fig. 3a), which are probably driven by local wind and sea-ice forcing that modifies the ocean density and temperature structure^30,33^ and generates eddies and internal waves that propagate into the TEIS cavity. During this period, thermal driving increased by 0.36 °C (Extended Data Fig. 2a), although a large proportion can be associated with the ever-increasing distance between the ocean mooring and the ice–ocean interface that results from basal melting (see Methods). At the same time, glacial meltwater concentration increased from about 11.0 ml l ^−1^ to about 13.4 ml l ^−1^ (Fig. 3b).

**Extended Data Fig. 3:**
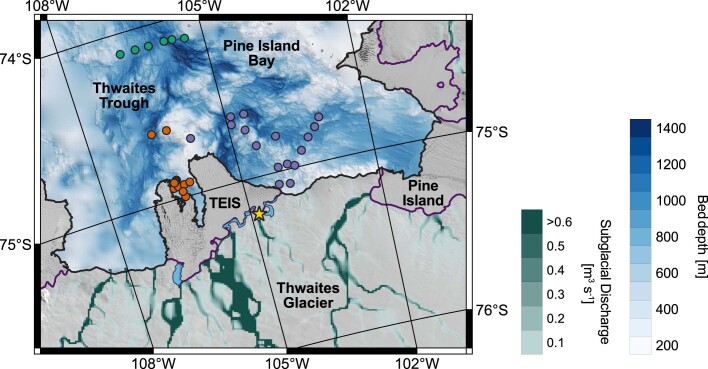
Subglacial discharge beneath Thwaites Glacier. Landsat 8 satellite image of Thwaites Glacier and the location of the hot-water-drilled access hole (yellow star; 75.207° S, 104.825° W) in the grounding-zone ‘butterfly’ region of TEIS. White–blue-coloured contours with hillshade show bed depth in the Amundsen Sea from ship-based survey^49^ and BedMachine^5^, whereas green-coloured contours show subglacial freshwater pathways and rate of discharge^39^. The purple, green and orange dots show the location of 2019–2020 ship-based CTD profiles from the International Thwaites Glacier Collaboration TARSAN project. The coastline (black) and grounding line (purple) are from the SCAR Antarctic Digital Database^50^. The blue-shaded area shows the location of the 2016–2017 grounding-zone region^18^. Extended Data Figure 3 was created with the QGIS Geographic Information System.

From September 2020, *S*_A_ begins to fall, whereas *Θ* remains constant at −0.2 °C (Fig. 3a). In *Θ*–*S*_A_ space, the hydrographic properties evolve along a horizontal trajectory, which cannot be explained by a change in source water mass (as meltwater mixing lines no longer intersect with the mCDW–WW thermocline). Instead, this trajectory is indicative of freshwater sourced from subglacial discharge at the grounding line (Fig. 3b,d). Subglacial waters beneath Thwaites come from basal melting of grounded ice that results from fast ice flow and large basal shear stress^34^. A persistent hydrological system exists upstream of Thwaites’ grounding line^35^ (Extended Data Fig. 3), along with subglacial lakes that exhibit episodic draining and filling events^36,37^. This hydrological system constantly reconfigures as a result of changes in the volume of meltwater production and glacier dynamics, and facilitates a flow of meltwater towards the grounding line in a channelized network, where it is discharged at the pressure-dependent freezing point^38,39^ (Extended Data Fig. 3). Although we cannot explain the mechanisms responsible for controlling discharge events, sedimentary evidence indicates that subglacial discharge beneath TEIS tends to occur in pulses^35^, consistent with the sudden onset we observe (Fig. 3b). Possible mechanisms include a shift in the drainage network to favour discharge beneath TEIS or the onset of a subglacial lake drainage event^36,37^. Subglacial discharge is linked to changes in basal friction and ice-stream velocity, and thus has the potential to modulate ice flow into the ocean^35,38^. In addition, subglacial freshwater input will drive a complex interplay between the density-driven enhancement in sub-ice-shelf circulation that should drive stronger basal melting and strengthening boundary-layer stratification that should suppress basal melting.

**Extended Data Fig. 4:**
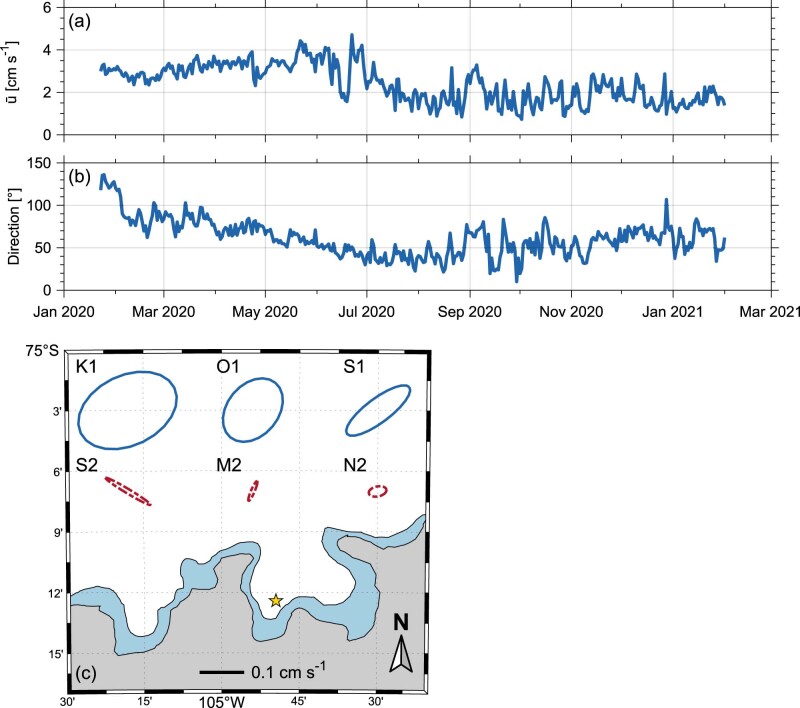
Flow speed, direction and tidal ellipses. **a**,**b**, Daily averaged time series of flow speed (**a**) and geographic flow direction (**b**) from the current meter deployed about 1.5 m beneath the ice base in the grounding-zone region of Thwaites Glacier (yellow star in Fig. 1). For flow direction, 0° indicates flow to the north, 90° indicates flow to the east and 150° indicates flow to the south of southeast. **c**, Amplitude and geographic orientation of the main diurnal (blue) and semidiurnal tidal constituents at the location of the borehole (yellow star). Solid lines indicate ellipses with positive semi-minor axes (anticlockwise rotation in time), whereas dot-dashed lines indicate ellipses with negative semi-minor axes (clockwise rotation in time). The blue polygon shows the location of the 2016–2017 grounding-zone region^18^, and the grey area shows where the ice is grounded. The map in panel **c** was created with MATLAB.

Ice base current speeds are key to setting basal melt rates. At our site, flow speeds are weak, averaging 2.4 cm s^−1^ (Fig. 4a,b and Extended Data Fig. 4a). Tidal variability is limited and is dominated by diurnal constituents (Extended Data Fig. 4c and Extended Data Table 1). Flow is oriented parallel to the grounding line (Fig. 4), with cooler and fresher meltwater-laden waters flowing towards the east in the upper layer, whereas warmer and saltier mCDW-derived waters flow to the west in the lower layer. The flow direction in the butterfly region is heavily steered by topography and is not necessarily representative of the westwards flow generally expected beneath TEIS^22,28^.

The magnitude of velocity, heat and salt mixing in the ice shelf–ocean boundary layer is challenging to measure and contributes substantial uncertainty when modelling the future behaviour of the Antarctic ice sheet^6^. Here we can indirectly derive the first estimate of the eddy viscosity beneath TEIS by examining the Ekman boundary layer that forms at the ice base (see Methods). Under the influence of rotation and frictional stresses, the flow direction observed by Icefin turns progressively clockwise as the boundary is approached, generating a transverse flow at the ice base (Fig. 4c). Fitting an analytical model for an under-ice Ekman boundary layer to the acoustic Doppler current profiler (ADCP) data yields an eddy viscosity of around 9 × 10^−4^ ± 5 × 10^−4^ m^2^ s^−1^, with an Ekman depth of about 3.6 m (Fig. 4c). This same Ekman behaviour is observed by the borehole current meter but it emerges as a function of time as the distance between the ice base and the instrument increases as the basal ice melts. The flow direction persistently swings anticlockwise from southeast in January 2020 to northeast in August 2020 (Fig. 3e and Extended Data Fig. 4b), after which the depth of the instrument exceeds the Ekman depth and the flow direction is no longer set primarily by distance from the boundary.

## Ice-shelf basal melting

**Extended Data Fig. 5:**
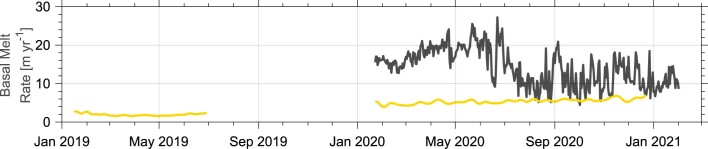
Extended ApRES basal melt-rate time series from the borehole location. Observed ApRES basal melt rate (yellow) at the borehole location (2020; yellow star in Fig. 1) and a secondary location 360 m downstream of the borehole location (2019; yellow diamond in Fig. 1) low-pass-filtered with a 15-day cutoff plotted against the basal melt rate estimated from the three-equation melt-rate model (grey; see Methods).

Despite the high thermal driving (Extended Data Fig. 2a), basal melt rates average no more than 2.0–5.4 m year^−1^ (Fig. 3c; see Methods). Low rates of basal melting have been observed since at least 2019 (Extended Data Fig. 5) and have probably persisted for much longer based on likely trends in ocean conditions (discussed in more detail later). The basal melt rate varies between sites and gradually increases with time; it is also no higher at the grounding line itself, as evidenced by an ApRES instrument that crossed from grounded to floating ice in April 2020 (orange curve in Fig. 3c). Spatial variations in the melt rate are probably associated with local flow patterns and variability in ice-shelf basal topography, as well as proximity to the grounding line, where the thinner, frictionally controlled water column with lower current speeds (Fig. 4) and weaker thermal driving^20^ (Fig. 2c) restricts basal melting.

Basal melting, controlled by the rate at which turbulent ocean mixing transports heat and salt vertically to the ice-shelf base through the ice shelf–ocean boundary layer^40^, is highly suppressed beneath TEIS by the strong stratification and quiescent ocean environment. Different boundary-layer turbulence regimes have been identified depending on the relative strength of the vertical current shear and buoyancy forcing: well-mixed shear controlled, stratified buoyancy controlled and diffusive-convective^8,41^. As the boundary layer beneath TEIS is characterized by weak current speeds (Fig. 4) and strong stratification (Fig. 2a), the well-mixed shear-controlled regime is precluded^42^. The temperature gradient is too weak to sustain strong diffusive-convective turbulence^26^ (Extended Data Fig. 1b) and thus the transport of heat through the boundary layer is predominantly controlled by stratified turbulence dynamics^7,8^. In this regime, weak flow speeds cannot generate sufficient shear-driven turbulence to overcome the highly stable ice-base stratification, strongly suppressing the vertical heat (and salt) transport to the ice base and, ultimately, the basal melt rate, despite strong thermal forcing^7^ (Fig. 2a). In the buoyancy-controlled regime beneath TEIS, basal melting is largely rate-limited by the density stratification and current speed, which control heat transport to the ice base, rather than by the amount of heat available. TEIS already exhibits excess levels of thermal driving (that is, there is more ocean heat available than that required to maintain basal melting) and the temperature increase required to drive substantially higher basal melt rates is probably unfeasible. Instead, order-of-magnitude increases in basal melting will only be driven by a large-scale acceleration in ocean circulation or a marked weakening of the ice-base stratification.

The canonical three-equation model for ice-shelf basal melting (see Methods) is widely used to generate sea-level projections, yet it is formulated exclusively for the well-mixed turbulence regime, in which the melt rate depends solely on the product of the thermal driving and flow velocity^40^. This formulation is not appropriate for TEIS. When forced with observed current speeds and thermal driving, it predicts melt rates upward of 14 m year^−1^, with a maximum of 32 m year^−1^, often exceeding observed values by more than an order of magnitude (Fig. 3c). This discrepancy arises as the model approximates the turbulent transfer of heat and salt through the ice shelf–ocean boundary layer using transfer coefficients that assume no influence of stratification (see Methods). Therefore, in the stratified regime, it substantially overestimates the efficiency of heat and salt transport through the boundary layer, and thus over-predicts the magnitude of the basal melt rate^7^ (Fig. 3c). Furthermore, this incorrect dependence on thermal driving and flow velocity means that the three-equation model as conventionally formulated cannot simulate the observed variability, predicting a fall in basal melt rates from May 2020 onwards owing to weaker current speeds, in contrast to the observations (Fig. 3c and Extended Data Fig. 4a). Although the transfer coefficients in the three-equation model could be reformulated to include some functional dependence on stratification, ultimately, knowledge of the vertical structure of density and velocity through the ice shelf–ocean boundary layer, knowledge that is widely lacking at present, must be incorporated into more sophisticated parameterizations to accurately predict melt rates under stratified conditions^7,8^.

**Extended Data Fig. 6:**
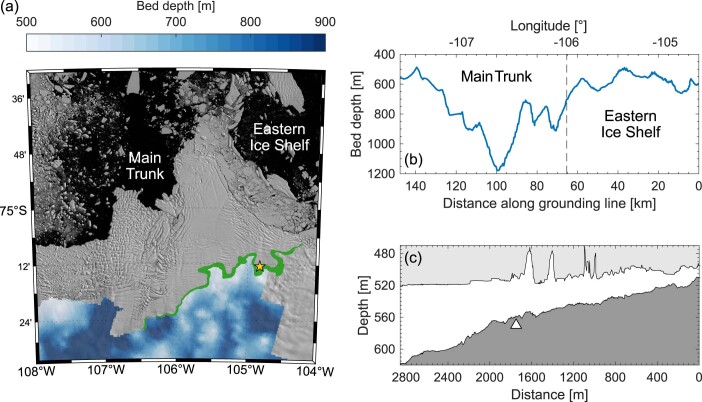
Bedrock depth along the Thwaites Glacier grounding line. **a**, Sentinel-2 image of TEIS and Thwaites Main Trunk from 9 February 2019. Coloured contours show gridded bed depth beneath present-day grounded ice from NASA IceBridge Multichannel Coherent Radar Depth Sounder (MCoRDS) data^68^ collected over Thwaites Glacier between 1 January 2006 and 31 December 2012. The green area indicates the location of the 2016–2017 grounding-zone region^18^, whereas the yellow star indicates the location of the hot-water-drilled access hole. **b**, Profile of bed depth along the present-day grounding line beneath TEIS and Thwaites Main Trunk from NASA IceBridge MCoRDS data^68^. The dotted line at 106° W marks the boundary between TEIS and the Thwaites Main Trunk. **c**, Profiles of ice base (light grey) and seabed (dark grey) from the Icefin T1 transect. The white triangle marks the location of the 2011 grounding line from satellite interferometry^18^. The map in panel **a** was created with MATLAB.

Much of the present-day grounding line beneath TEIS sits on a bedrock ridge that runs northeast to southwest beneath the ice shelf (Extended Data Fig. 6a). Bed depth along the grounding line is relatively constant and generally no deeper than that in the butterfly region (Extended Data Fig. 6b). Basal melt rates throughout much of the TEIS grounding zone are therefore unlikely to be substantially higher than our observed value. The weak basal melt conditions observed here contrast with numerical models^28,43^, which suggest that TEIS grounding-zone basal melt rates are an order of magnitude higher. We note that the modest melt rates we observe are not representative of the main trunk of Thwaites Glacier, which is characterized by much steeper basal slope angles and is grounded on bedrock >1,000 m below sea level (Extended Data Fig. 6b). As such, basal melting of the main trunk would be expected to be very much higher^18,44^.

Despite weak basal melting throughout the TEIS grounding zone, the grounding line has retreated rapidly at a rate of 0.6–1.2 km year^−1^ between 2011 and 2017 (ref. ^18^). Although the retreat rate is spatially variable, the grounding line has continued to retreat over the period covered by our melt rate observations (2019–2021), widely reaching 0.4 km year^−1^ throughout the butterfly region, with a maximum >1.5 km year^−1^ (Fig 1). Thus our observations suggest that the rapid grounding-line retreat beneath TEIS since 2011 has probably been associated with relatively modest basal melt rates. Indeed, neither the increase in thermal forcing associated with the deeper 2011 grounding line (about 0.7 °C higher; Extended Data Fig. 6c) nor the interannual variability in thermocline depth in Pine Island Bay^30,31^ are sufficient to drive order-of-magnitude changes in basal melting, consistent with the stratified turbulence regime. The strong stratification observed at the ice base that is responsible for suppressing the basal melt rate is probably highly persistent, maintained by the input of glacial meltwater and subglacial discharge (Fig. 3b). At the same time, there is little oceanographic evidence to suggest that current speeds would have been much higher in the past to erode this stratification, as the region is subject to weak tidal forcing, and narrow, frictionally controlled water columns close to grounding lines with a flat ice base are not conducive to rapid flow. However, coupled with melting in the vicinity of the seaward pinning point of TEIS^14,22^, even relatively modest basal melting in the grounding zone can still force notable change to grounded ice. A small increase in basal melting can create a large melt imbalance that triggers melt-induced thinning of TEIS and drives a reduction in basal drag at the grounding line^6,45^. The reduction in basal drag weakens the back stress imposed by the ice shelf^46^, resulting in a loss of buttressing and dynamic thinning of grounded ice upstream^47,48^. As this thinner ice goes afloat, the grounding line can retreat rapidly inland and up the prograde bedrock slope characteristic of the butterfly region. Although ice–ocean models suggest that high rates of basal melting beneath newly floating ice can provide a strong positive feedback to continuing retreat^6^, our results indicate that this feedback is weak. Nevertheless, sustained grounding-zone basal melting, weaker ice-shelf buttressing and the advection of increasingly thinner ice over the grounding line will continue to condition TEIS to persistent retreat in the future, even without a strong positive feedback from elevated basal melting^6^.

## Methods

### Borehole observations

A Sea-Bird Scientific SBE 49 FastCAT CTD profiler was used to observe the water-column structure between 9 January and 12 January 2020. A total of 15 individual CTD casts were completed, sampling at a rate of 16 Hz. Before each deployment, the CTD was stored in a warm bath (approximately 5 °C) to minimize icing in the conductivity cell during the profiler’s traverse of the approximately 90-m air-filled portion of the borehole. The CTD data were processed using standard routines in the Sea-Bird data-processing software version 7.26.7.129 and each profile was averaged into 0.1-m vertical bins. Absolute salinity and conservative temperature were computed using the Gibbs SeaWater (GSW) Oceanographic Toolbox for TEOS-10 (refs. ^53,54^). The temperature and conductivity sensors were manufacturer-calibrated before deployment and the stated accuracy of the sensors are ±0.002 °C and ±0.0003 S m^−1^, respectively.

A moored turbulence instrument cluster was deployed about 1.5 m beneath the ice base to observe the small-scale turbulent fluctuations in the ice shelf–ocean boundary layer^42^. Consisting of a Nobska Modular Acoustic Velocity Sensor (MAVS) differential acoustic travel-time 3D velocity sensor, an RBRcoda fast-response temperature sensor and an RBRconcerto inductive conductivity sensor, the turbulence instrument cluster was scheduled to operate in burst mode, sampling at 5 Hz for 15 min every 2 h. For each 15-min burst, the average temperature, conductivity and velocity values were received over an Iridium satellite link. In this study we use the mean values from 4,459 individual bursts collected between 23 January 2020 and 1 February 2021. An analysis of the full 5-Hz turbulence data awaits a future study. The stated uncertainty in the velocity components is 3 mm s^−1^, whereas the stated uncertainty in the temperature and conductivity data are ±0.002 °C and ±0.0003 S m^−1^, respectively. A small offset in the conductivity data caused by proximity effects associated with the inductive sensor was removed through regression against the CTD conductivity data.

### Lateral gradients in temperature and salinity

CTD profiling beneath TEIS was largely carried out in three separate sessions with approximately 19 h between the first and second sessions and 47 h between the second and third sessions. Dividing the mean absolute difference in temperature and salinity between each session pair by the lateral advective distance over the time separating each pair (assuming a mean flow speed of 3 cm s^−1^ during the CTD deployment period; Extended Data Fig. 4a) gives a mean lateral gradient of 2.7 × 10^−3^ ± 10^−4^ °C km^−1^ for temperature and 1.1 × 10^−3^ ± 10^−5^ g kg^−1^ km^−1^ for salinity. The effect of tidal flow has been ignored in these calculations, as the tidal flow speeds are an order of magnitude weaker.

### ApRES

An ApRES was established within 10 m of the borehole on 23 January 2020 and set to record a burst of 20 measurements once every 2 h. The data were recovered from the instrument on 27 December 2020. Data from four further ApRES deployments are also presented in this study (Fig. 1): a 5-month record from the first half of 2019 from an instrument deployed 360 m downstream of the borehole and data from three sites from 2020, contemporaneous with the borehole dataset. One of the 2020 sites was 1,310 m downstream of the borehole, another 1,340 m upstream of the borehole and, finally, one instrument was deployed on grounded ice 2,600 m upstream of the borehole, which tracked across the grounding line during 2020 and recorded every 3 h.

ApRES uses frequency-modulated continuous-wave modulation, with a chirp that scans from 200 to 400 MHz over a 1-s period. The measurements in each burst were checked for quality and then averaged. Each averaged burst was processed^55^ to generate a radar return that preserves the signal phase.

By using both amplitude and phase, ApRES can monitor the changing distance between the antennas and the ice-shelf base with millimetre-scale precision. This raw Lagrangian ice-shelf thinning includes both the basal melt signal and the ice-column vertical strain that results from ice flow and snow compaction^21^. As well as the range to the ice base, the range to reflecting horizons within the ice column can be monitored and used to estimate the vertical strain within the ice as follows. The motion of internal reflecting horizons in any given depth interval can be found by cross-correlating the complex return for sequential (two-hourly) measurements for the entire time series. The vertical motion of the ice within the layer from one return to the next was derived from the phase of the cross-correlation and the reliability of that estimate was indicated by its amplitude. As all displacements are measured with respect to the antennas, the vertical displacement of any individual layer is the effect of the integrated strain in the ice above. The ice-shelf thinning rate, as measured from the antennas, is obtained by using a depth interval that tracks the return from the ice base.

For tracking the ice base to obtain the total thinning rate, an assumption in ApRES data processing is that, over the period of the time series, the topography of the ice base local to the radar does not change at length scales at or longer than the wavelength of the radar waves in ice, in this case, at length scales greater than about 0.5 m. This requirement was not met at the borehole site nor at various periods of the time series from the upstream sites. However, it was possible to use the first multiple echo, which is the result of the radar signal travelling from the transmit antenna to the ice base, back to the ice surface and then back to the ice base, before finally returning to the receive antenna. The range to the multiple is largely immune to local topographic evolution in the ice base, presumably because of the much larger effective footprint. The multiple is very much weaker than the first basal return, but its phase can be reliably tracked. For the downstream ApRES deployment, and those sections of the other deployments when the first basal return was not changing its form, the melt-rate time series from the first and second returns yielded a satisfactory match. Short-term variations in derived melt rate (3 to 5 days) were much stronger from the multiple, possibly resulting from snow-accumulation events.

Vertical profiles of vertical ice velocities, averaged across the entire time series, were calculated by dividing the return into 4-m layers, cross-correlating as described above and calculating the mean vertical velocity for each layer. From these profiles a depth interval was selected from the lower part of the ice column from which to calculate the integrated non-melt contribution to the thinning rate. The selection of the depth interval was based on the strength of the time-averaged correlations and, for the downstream site, the borehole site and the first upstream site, was 300–472 m, 304–436 m and 300–550 m, respectively. The strain from one measurement to the next was averaged across the depth interval and the time series differentiated and low-pass-filtered to provide a time series of vertical velocity variability at timescales of 5 days and longer. Although the strain rate in the ice column was expected to evolve slowly as the ice moved downstream, we assumed that non-tidal short-term variations would not be present. The vertical profiles of vertical velocity showed an approximately linear gradient through the selected depth intervals and an offset vertical velocity was determined by extrapolating that variation to the depth of the basal reflector. That offset was then added to the vertical velocities to yield the final non-melt contribution to the time series of ice-shelf thinning rate. The 2019 site, and the initially grounded site, were processed slightly differently. The internal reflections from near the ice base were good enough to allow a deep depth interval to be selected and cross-correlated to yield an integrated vertical strain directly. Intervals from 520 to 580 m and from 515 to 550 m were used for the 2019 and initially grounded sites, respectively, with a minor correction to accommodate a small, approximately linearly increasing strain rate near the base.

The final melt-rate time series was calculated by subtracting the non-melt contribution from the thinning rate and then low-pass-filtering at a cutoff of 15 days.

### Icefin remotely operated underwater vehicle

Icefin was equipped with a Neil Brown Ocean Sensors conductivity–temperature (CT) sensor and a Valeport ultraP pressure sensor. The stated manufacturer accuracies are ±0.001 S m^−1^, ±0.005 °C and 0.1 dbar for conductivity, temperature and pressure, respectively, which translate into uncertainties of ±0.008 g kg^−1^ for *S*_A_ and ±0.018 °C for *Θ*. All sensors were factory calibrated before deployment and then cross-compared with the SBE 49 CTD profiler to remove offsets in conductivity and temperature of 0.0286 S m^−1^ and 0.0236 °C. The CT sensor recorded at a frequency of 5 Hz, whereas the pressure sensor recorded at 1 Hz. Pressure measurements were interpolated to match the 5-Hz CT data. Hydrographic data were post-processed by removing outliers that exceeded more than two standard deviations from the mean, as well as data points collected when the vehicle speed was lower than 5 cm s^−1^. A three-point weighted-mean filter was also applied to the conductivity and temperature data.

Ocean current speeds were measured using a LinkQuest NavQuest 600 Micro Doppler Velocity Log, which doubles as an ADCP. The ADCP provides measurements of the current speed in 2-m bins at a variable distance from the vehicle, controlled by gradients in the pitch, roll, heading and speed of the vehicle. Uncertainty in the current velocity is typically 1% of the vehicle’s velocity in its direction of travel. As Icefin travels at speeds ≤50 cm s^−1^, the uncertainty in velocity recorded by the ADCP in the direction of travel is ≤5 mm s^−1^. The uncertainty in velocities perpendicular to the direction of travel is typically much lower. The velocity data were recorded at a rate of 1 Hz and were post-processed by removing data points when the vehicle pitch or roll is greater than 30°. A 30-s running mean filter was applied to all data points and measurements were filtered for gradients greater than one standard deviation from the mean in vehicle speed, pitch, roll and individual bin velocity. Finally velocities were bin-averaged into 1-m depth bins and velocities were excluded if they exceeded one standard deviation of the mean for each bin.

### Ship-based CTD profiles

A dual-sensors system based on a Sea-Bird 911 CTD was used for conductivity, temperature and pressure measurements outside the ice-shelf cavity from the RVIB Nathaniel B. Palmer in 2019 and 2020. Standard Sea-Bird software Seasave version 7.26.1.8 was used for data collection and conductivity cell thermal mass correction in 2019 and Seasave version 7.26.7.121 in 2020. Manufacturer-recommended values for cell thermal mass correction were used as follows: thermal anomaly amplitude, *α* = 0.03 and thermal anomaly time constant 1/*β* = 7.0. Water samples were taken from the CTD rosette and analysed using a Guildline Portasal salinometer to calibrate the primary and secondary conductivity sensors on the CTD profiler.

### Density ratio and Turner angle

Double-diffusive convection occurs as a result of the difference in molecular diffusivities between salt and heat^56^. Under Antarctic ice shelves, the presence of cold and fresh meltwater-laden waters above warm and salty modified Circumpolar Deep Water drives a double-diffusive process known as diffusive convection. Strong diffusive convection can lead to the formation of ‘diffusive staircases’, where well-mixed layers in temperature and salinity are separated by sharp interfaces^57,58^. Diffusive convection can still occur without staircase formation however. Diffusive convection can exert a first-order control on the rate of ice-shelf basal melting^59,60^.

The susceptibility of a water column to diffusive convection can be characterized through the density ratio

1 $${R}_{{\rho }}={\alpha }\frac{\partial \varTheta }{\partial {\rm{z}}}/{\beta }\frac{\partial {S}_{{\rm{A}}}}{\partial {\rm{z}}},$$

which measures the degree of compensation between temperature and salinity gradients in terms of their effect on density stratification. *α* is the thermal expansion coefficient, *β* the haline contraction coefficient, $\frac{\partial \varTheta }{\partial {\rm{z}}}$ the vertical gradient of conservative temperature and $\frac{\partial {S}_{{\rm{A}}}}{\partial \text{z}}$ is the vertical gradient of absolute salinity. A water column is susceptible to diffusive convection when *R*_*ρ*_ is between 0 and 1, with the strength of diffusive convection increasing as *R*_*ρ*_ approaches 1. The Turner angle

2 $${\rm{Tu}}=\arctan 2\left({\alpha }\frac{\partial \varTheta }{\partial {\rm{z}}}+{\beta }\frac{\partial {S}_{{\rm{A}}}}{\partial {\rm{z}}},{\alpha }\frac{\partial \varTheta }{\partial {\rm{z}}}-{\beta }\frac{\partial {S}_{{\rm{A}}}}{\partial {\rm{z}}}\right)$$

is related to the density ratio, in which arctan2 is the four-quadrant inverse tangent (tan^−1^) and the water column is susceptible to diffusive convection when Tu is between −45° and −90°.

### Glacial meltwater and subglacial discharge fractions

**Extended Data Fig. 7:**
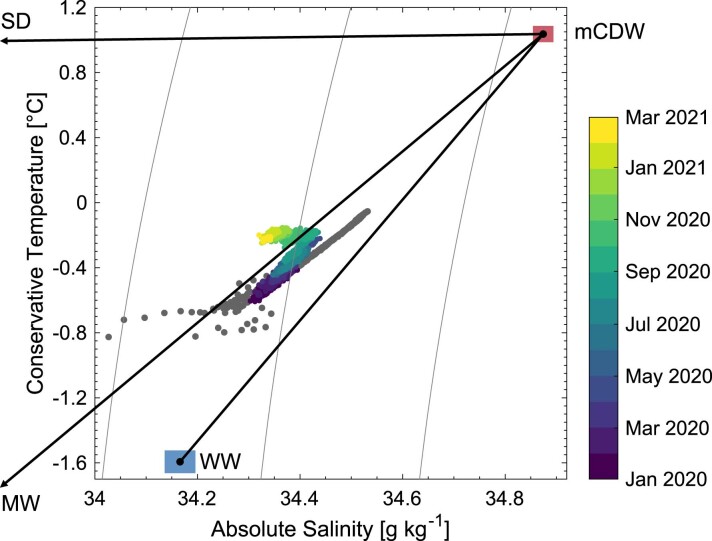
Temperature–salinity diagram and linear mixing lines between the main sub-ice shelf water masses. Linear mixing lines between the mCDW (red box), WW (blue box), glacial MW and SD, along with *Θ*–*S*_A_ observations from each individual CTD cast (grey dots) and the sub-ice shelf mooring (dots coloured by time). Note that the endmember properties of MW and SD fall outside the range of the axes (Extended Data Table 2). From the end of September 2020 onwards, individual *Θ*–*S*_A_ observations lie above the mCDW–MW mixing line, indicating the presence of subglacial discharge and the negligible influence of WW.

Meltwater fractions are calculated using the composite tracer method^27^ and water-mass endmembers derived from the ship-based CTD profiles. In the absence of glacial meltwater or subglacial discharge, it is assumed that the ambient water column beneath TEIS would be composed exclusively of mCDW and WW that mix along a straight line between their corresponding endmembers^61^ (Fig. 2c and Extended Data Fig. 7). For each conservative temperature and absolute salinity observation, a composite tracer can be constructed

3 $${\psi }_{{{\rm{o}}{\rm{b}}}_{{\rm{W}}{\rm{W}}}}=({\Theta }_{{\rm{m}}{\rm{C}}{\rm{D}}{\rm{W}}}-{\Theta }_{{\rm{o}}{\rm{b}}})-({S}_{{{\rm{A}}}_{{\rm{m}}{\rm{C}}{\rm{D}}{\rm{W}}}}-{S}_{{{\rm{A}}}_{{\rm{o}}{\rm{b}}}})\left(\frac{{\Theta }_{{\rm{m}}{\rm{C}}{\rm{D}}{\rm{W}}}-{\Theta }_{{\rm{W}}{\rm{W}}}}{{S}_{{{\rm{A}}}_{{\rm{m}}{\rm{C}}{\rm{D}}{\rm{W}}}}-{S}_{{{\rm{A}}}_{{\rm{W}}{\rm{W}}}}}\right),$$

in which *Θ*_mCDW_ and ${S}_{{{\rm{A}}}_{{\rm{mCDW}}}}$ are the conservative temperature and absolute salinity of the mCDW endmember, respectively, *Θ*_WW_ and ${S}_{{{\rm{A}}}_{{\rm{WW}}}}$ are the conservative temperature and absolute salinity of the WW endmember, respectively, and *Θ*_ob_ and $\,{S}_{{{\rm{A}}}_{{\rm{ob}}}}$ are observed values of conservative temperature and absolute salinity, respectively. If a data point lies on the ambient mCDW–WW mixing line, ${\psi }_{{{\rm{o}}{\rm{b}}}_{{\rm{W}}{\rm{W}}}}$ is equal to zero. The value of ${\psi }_{{{\rm{o}}{\rm{b}}}_{{\rm{W}}{\rm{W}}}}$ will become non-zero, however, if glacial meltwater (MW) causes a data point to move off the ambient mixing line. The value of the composite tracer in pure MW is

4 $${\psi }_{{{\rm{M}}{\rm{W}}}_{{\rm{W}}{\rm{W}}}}=({\Theta }_{{\rm{m}}{\rm{C}}{\rm{D}}{\rm{W}}}-{\Theta }_{{\rm{M}}{\rm{W}}})-({S}_{{{\rm{A}}}_{{\rm{m}}{\rm{C}}{\rm{D}}{\rm{W}}}}-{S}_{{{\rm{A}}}_{{\rm{M}}{\rm{W}}}})\left(\frac{{\Theta }_{{\rm{m}}{\rm{C}}{\rm{D}}{\rm{W}}}-{\Theta }_{{\rm{W}}{\rm{W}}}}{{S}_{{{\rm{A}}}_{{\rm{m}}{\rm{C}}{\rm{D}}{\rm{W}}}}-{S}_{{{\rm{A}}}_{{\rm{W}}{\rm{W}}}}}\right),$$

in which *Θ*_MW_ and ${S}_{{{\rm{A}}}_{{\rm{MW}}}}$ are the conservative temperature and absolute salinity of the MW endmember, respectively. The fraction of glacial meltwater (*x*_MW_) present in the water column can then be calculated from

5 $${x}_{{\rm{M}}{\rm{W}}}=\frac{{\psi }_{{{\rm{o}}{\rm{b}}}_{{\rm{W}}{\rm{W}}}}}{{\psi }_{{{\rm{M}}{\rm{W}}}_{{\rm{W}}{\rm{W}}}}}.$$

To quantify the variability in *x*_MW_ caused by uncertainty in the water-mass endmembers, 1,000 independent estimates of the meltwater fraction were made using a set of random endmember properties derived from the normal distribution described by the mean and standard deviation of each endmember property (Extended Data Table 2). The observed meltwater fraction is given by the mean of the 1,000 independent estimates, and the uncertainty is given by the standard error of the mean. In general, the uncertainty is two orders of magnitude smaller than the mean.

The conservative temperature and absolute salinity of the mCDW and WW endmembers were extracted from the ship-based CTD casts collected in front of TEIS (Fig. 2c). The properties of the WW endmember (Extended Data Table 2) are set to those found at the depth of the temperature minimum below the surface layer, whereas the mCDW endmember properties are set to those found at the depth of the temperature maximum. The MW endmember has an effective conservative temperature of −90.8 °C (ref. ^27^) and an absolute salinity of 0 g kg^−1^, whereas the conservative temperature of the subglacial discharge endmember is set to the pressure-dependent in situ freezing temperature for freshwater at the depth of the grounding line (−0.36 °C) with an absolute salinity of 0 g kg^−1^. The endmember values are consistent with those used in previous studies^61,62^.

Starting in September 2020, a persistent signal of fresh subglacial discharge (SD) appears in the hydrographic data. In *Θ*–*S*_A_ space, individual data points fall above the mCDW–MW mixing line, indicative of the presence of this fourth water mass (Extended Data Fig. 7). With only *Θ* and *S*_A_ available as tracers, it is not possible to solve for all water-mass fractions simultaneously, as the system is underdetermined. Instead, we have to make a necessary assumption that the influence of WW is negligible and that the water column is composed solely of a mix of mCDW, MW and SD. Although this practical assumption cannot be fully justified, WW is typically found above a depth of 400 m in the Amundsen Sea^61,63,64^ and is therefore mostly excluded from the grounding-zone region owing to the depth of the ice base (Fig. 4). As a result, the impact of this assumption on the water-mass fractions is probably small. To determine the SD faction for data points that lie outside the mCDW–WW–MW mixing triangle, a composite tracer is constructed that is equal to zero for data points that lie along the mCDW–MW mixing line:

6 $${\psi }_{{{\rm{ob}}}_{{\rm{MW}}}}=\left({\varTheta }_{{\rm{mCDW}}}-{\varTheta }_{{\rm{ob}}}\right)-\left({S}_{{{\rm{A}}}_{{\rm{mCDW}}}}-{S}_{{{\rm{A}}}_{{\rm{ob}}}}\right)\left(\frac{{\varTheta }_{{\rm{mCDW}}}-{\varTheta }_{{\rm{MW}}}}{{S}_{{{\rm{A}}}_{{\rm{mCDW}}}}-{S}_{{{\rm{A}}}_{{\rm{MW}}}}}\right).$$

The value of this composite tracer in pure SD is

7 $${\psi }_{{\rm{SD}}}=\left({\varTheta }_{{\rm{mCDW}}}-{\varTheta }_{{\rm{SD}}}\right)-\left({S}_{{{\rm{A}}}_{{\rm{mCDW}}}}-{S}_{{{\rm{A}}}_{{\rm{SD}}}}\right)\left(\frac{{\varTheta }_{{\rm{mCDW}}}-{\varTheta }_{{\rm{MW}}}}{{S}_{{{\rm{A}}}_{{\rm{mCDW}}}}-{S}_{{{\rm{A}}}_{{\rm{MW}}}}}\right),$$

in which *Θ*_SD_ and ${S}_{{{\rm{A}}}_{{\rm{SD}}}}$ are the conservative temperature and absolute salinity of the SD endmember, respectively, and the SD fraction can be calculated from

8 $${x}_{{\rm{SD}}}=\frac{{\psi }_{{{\rm{ob}}}_{{\rm{MW}}}}}{{\psi }_{{\rm{SD}}}}.$$

Similarly, the MW fraction for data points that lie outside the mCDW–WW–MW mixing triangle can be derived by constructing a composite tracer that is equal to zero for data points that lie along the mCDW–SD mixing line:

9 $${\psi }_{{{\rm{ob}}}_{{\rm{SD}}}}=\left({\varTheta }_{{\rm{mCDW}}}-{\varTheta }_{{\rm{ob}}}\right)-\left({S}_{{{\rm{A}}}_{{\rm{mCDW}}}}-{S}_{{{\rm{A}}}_{{\rm{ob}}}}\right)\left(\frac{{\varTheta }_{{\rm{mCDW}}}-{\varTheta }_{{\rm{SD}}}}{{S}_{{{\rm{A}}}_{{\rm{mCDW}}}}-{S}_{{{\rm{A}}}_{{\rm{SD}}}}}\right).$$

Taking the value of this composite tracer in pure MW as

10 $${\psi }_{{{\rm{M}}{\rm{W}}}_{{\rm{S}}{\rm{D}}}}=({\Theta }_{{\rm{m}}{\rm{C}}{\rm{D}}{\rm{W}}}-{\Theta }_{{\rm{M}}{\rm{W}}})-({S}_{{{\rm{A}}}_{{\rm{m}}{\rm{C}}{\rm{D}}{\rm{W}}}}-{S}_{{{\rm{A}}}_{{\rm{M}}{\rm{W}}}})\left(\frac{{\Theta }_{{\rm{m}}{\rm{C}}{\rm{D}}{\rm{W}}}-{\Theta }_{{\rm{S}}{\rm{D}}}}{{S}_{{{\rm{A}}}_{{\rm{m}}{\rm{C}}{\rm{D}}{\rm{W}}}}-{S}_{{{\rm{A}}}_{{\rm{S}}{\rm{D}}}}}\right),$$

the MW fraction can be calculated as

11 $${x}_{{\rm{M}}{\rm{W}}}=\frac{{\psi }_{{{\rm{o}}{\rm{b}}}_{{\rm{S}}{\rm{D}}}}}{{\psi }_{{{\rm{M}}{\rm{W}}}_{{\rm{S}}{\rm{D}}}}}.$$

### Ice base Ekman boundary layer

Assuming a momentum balance between a steady, uniform, geostrophic flow beneath a flat ice base and the frictional stress exerted by the ice base against this flow^65,66^:

12 $$-fv=-\frac{1}{{\rho }_{0}}\frac{\partial p}{\partial x}+\nu \frac{{\partial }^{2}u}{\partial {z}^{2}}$$

13 $$+fu=-\frac{1}{{\rho }_{0}}\frac{\partial p}{\partial y}+\nu \frac{{\partial }^{2}v}{\partial {z}^{2}}$$

14 $$0=-\frac{1}{{\rho }_{0}}\frac{\partial p}{\partial z},$$

in which *f* is the Coriolis parameter, *ρ*_0_ is the fluid density, *p* is pressure, *ν* is the kinematic eddy viscosity and *u* and *v* are the horizontal velocity components, then the vertical structure of the horizontal velocity components through the boundary layer are given by the canonical Ekman solution^66^

15 $$u={u}_{{\rm{g}}}\left(1-{{\rm{e}}}^{-z/d}\cos \frac{z}{d}\right)$$

16 $$v={u}_{{\rm{g}}}{{\rm{e}}}^{-z/d}\sin \frac{z}{d},$$

in which *u*_g_ is the magnitude of the far-field geostrophic flow, *z* is boundary layer depth and *d* is the Ekman depth:

17 $$d=\sqrt{\frac{2\nu }{f}}.$$

Using the root mean square error as a cost function, we fit equations (15) and (16) to the *u* and *v* boundary-layer velocity profiles from Icefin to determine the value of the eddy viscosity and the Ekman depth beneath TEIS.

### Thermal driving and the three-equation model for basal melting

The rate of ice-shelf basal melting is controlled by the divergence of the sensible heat flux at the phase change interface^40^

18 $${\rho }_{{\rm{i}}}{a}_{{\rm{b}}}{L}_{{\rm{i}}}={{\rho }_{{\rm{i}}}{c}_{{\rm{i}}}{\kappa }_{{\rm{i}}}\frac{{\rm{\partial }}{T}_{{\rm{i}}}}{{\rm{\partial }}z}|}_{{\rm{b}}}+{\rho }_{{\rm{w}}}{c}_{{\rm{w}}}\langle {W}^{{\prime} }{T}_{w}^{{\prime} }\rangle ,$$

in which *ρ* is density, *a*_b_ the basal melt rate, *L*_*i*_ the latent heat of fusion of ice, *c* the specific heat capacity, *κ*_*i*_ the thermal diffusivity of ice, *T* the temperature and *W* is the vertical ocean velocity. The subscripts i, b and w refer to ice, ice–ocean boundary and ocean, respectively. The primes refer to turbulent fluctuations and the angled brackets to the time average. The first term on the right-hand side is the conductive heat flux into the ice, whereas the second term represents the vertical turbulent heat flux through the oceanic boundary layer. In the absence of direct turbulence measurements, or in regional or global models that do not resolve the vertical scales of the ice shelf–ocean boundary layer, the second term is quantified through a simple turbulence closure scheme that models the heat flux, $\langle {W}^{{\prime} }{T}_{w}^{{\prime} }\rangle$, as a product of the drag coefficient (*C*_d_), a non-dimensional turbulent transfer coefficient for temperature (*Γ*_T_), the horizontal ocean velocity (*U*), and the thermal driving (*T*_d_), that is given as the difference between the in situ ocean temperature some distance from the ice–ocean boundary (*T*_w_; in this case, the temperature at the ocean mooring deployed 1.5 m beneath the ice shelf) and the temperature at the ice–ocean boundary (*T*_b_) that is assumed to be at the in situ freezing point at salinity *S*_b_ and pressure *P*_b_:

19 $${\rho }_{{\rm{w}}}{c}_{{\rm{w}}}\langle {W}^{{\prime} }{T}_{w}^{{\prime} }\rangle ={\rho }_{{\rm{w}}}{c}_{{\rm{w}}}{C}_{{\rm{d}}}^{1/2}{\varGamma }_{{\rm{T}}}U[{T}_{{\rm{w}}}-\,{T}_{{\rm{b}}}({S}_{{\rm{b}}},{P}_{{\rm{b}}})].$$

The in situ freezing point at the ice–ocean boundary is given by the liquidus condition

20 $${T}_{{\rm{b}}}={\lambda }_{1}{S}_{{\rm{b}}}+{\lambda }_{2}+{\lambda }_{3}{P}_{{\rm{b}}},$$

in which *λ*_1_, *λ*_2_ and *λ*_3_ are constants and the boundary salinity (*S*_b_) is given by the salt balance at the phase-change interface:

21 $${\rho }_{{\rm{i}}}{a}_{{\rm{b}}}\left({S}_{{\rm{b}}}-{S}_{{\rm{i}}}\right)={\rho }_{{\rm{w}}}{C}_{{\rm{d}}}^{1/2}{\varGamma }_{{\rm{S}}}U\left[{S}_{{\rm{w}}}-{S}_{{\rm{b}}}\right],$$

in which *Γ*_S_ is the turbulent transfer coefficient for salt and the salinity of ice (*Si*) is taken to be zero for ice shelves. The turbulent transfer coefficients, *Γ*_T_ and *Γ*_S_, assume that the thermal and saline diffusive sublayers (diffusive regions next to the ice base that are dominated by molecular-scale processes) are controlled exclusively by current shear and thin with increasing current velocity; however, there are known to be many ice shelf–ocean environments where this is not the case^8^. The system of equations described by equations (18)–(21) represents the canonical three-equation model for ice-shelf basal melting^40,67^. It can be solved for the basal melt rate given observed ocean temperature, salinity and flow speed and physical constants in Extended Data Table 3 (refs. ^40,42^).

Because thermal driving is defined as the difference between the ocean temperature recorded by the mooring and the freezing temperature at the ice–ocean boundary, its magnitude is sensitive to the distance between the ocean mooring and the ice base. Basal melting increases the distance between the ocean mooring and the ice base as a function of time, and this drives an apparent increase in thermal driving without a change in the source water mass as the mooring effectively descends into warmer water. If we assume that the temperature profile through the boundary layer is fixed with respect to time, we can use the observed CTD profiles to estimate that the apparent change in thermal driving owing to ice-base recession is about 0.2 °C, or roughly 57% of the observed change (Extended Data Fig. 2a).

Although the rate of basal melting beneath TEIS predicted by the three-equation melt-rate model is linearly related to the magnitude of the thermal driving, it is relatively insensitive to the apparent change in thermal driving owing to ice-base recession. Indeed, the mean predicted melt rate only falls by 8% when thermal driving is corrected for ice-base recession (Extended Data Fig. 2b) and the predicted melt rate remains an order of magnitude higher than the observed values. The small reduction in predicted melt rate is consistent with our assertion that basal melting beneath TEIS is limited by the strong stratification, weak flow speeds and the lack of shear-driven turbulence, rather than the amount of heat available in the boundary layer. Irrespective of ice-base recession, thermal driving continues to exceed 1.8 °C by the beginning of 2021 (Extended Data Fig. 2a), highlighting the substantial amount of heat that is available in the grounding-zone region to drive basal melting. The apparent increase in thermal driving owing to ice-base recession therefore has no impact on our conclusion that the three-equation melt-rate model is unable to accurately predict the melt rate beneath TEIS.

## Online content

Any methods, additional references, Nature Portfolio reporting summaries, source data, extended data, supplementary information, acknowledgements, peer review information; details of author contributions and competing interests; and statements of data and code availability are available at 10.1038/s41586-022-05586-0.

## Figures and Tables

**Extended Data Table 1:**
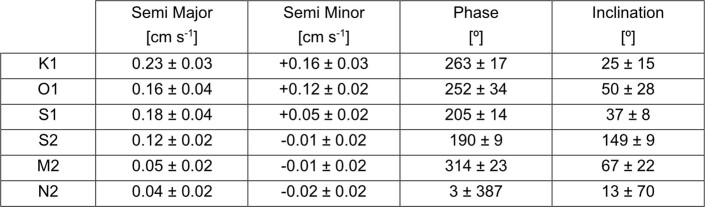
Tidal ellipse parameters for the six main tidal constituents

Ellipse parameters for the six strongest diurnal and semidiurnal tidal constituents in the grounding-zone region of Thwaites Glacier. Inclination angles are defined positive anticlockwise from due east. Values are presented as the mean ± 95% confidence intervals for each parameter determined through Monte Carlo uncertainty propagation.

**Extended Data Table 2:**
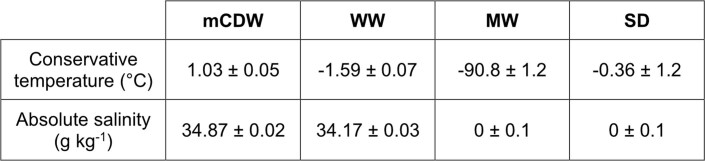
Temperature and salinity properties of the main sub-ice shelf water masses

Endmember values for the different water masses used in the water mass fraction calculations (see Methods). Values are presented as the mean ± standard deviation for each parameter.

**Extended Data Table 3:**
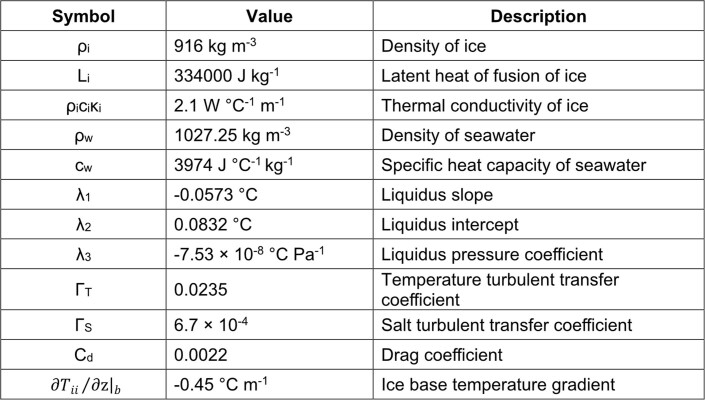
Physical constants used in the three-equation melt-rate model

Values of the physical constants used in the three-equation model for ice-shelf basal melting.

## Data Availability

The CTD data are available from the UK Polar Data Centre (10.5285/97204415-683f-4d55-8b38-a2700fa94efe). The mooring data are available from the UK Polar Data Centre (10.5285/4ffad557-1c3c-4ea7-a73d-6d782331b08a). The ApRES basal melt-rate data are available from the UK Polar Data Centre (10.5285/B81BFF87-3429-4754-B4B5-C6B58198E744). The Icefin data are available from the United States Antarctic Program Data Center (10.15784/601618).

## Code availability

The MATLAB Gibbs-SeaWater (GSW) Oceanographic Toolbox for TEOS-10 (v3.06) is available at: https://www.teos-10.org/software.htm. The custom MATLAB (R2021a) code and additional materials and libraries required to process the data and generate the figures in the article are available on request from the corresponding author.
